# Cognitive and Emotional Impairments in Acute Post-Stroke Patients—A Cross-Sectional Study

**DOI:** 10.3390/medicina61101739

**Published:** 2025-09-24

**Authors:** Maja Ibic, Lara Miklič, Sofia Rakusa, Jan Zmazek, Marija Menih, Kim Caf, Martin Rakusa

**Affiliations:** 1 Faculty of Medicine, University of Maribor, 2000 Maribor, Slovenia; 2 University Division of Neurology, University Medical Centre Maribor, 2000 Maribor, Slovenia; 3 National Institute of Public Health, 1000 Ljubljana, Slovenia; 4 Faculty of Natural Sciences and Mathematics, University of Maribor, 2000 Maribor, Slovenia; 5 University Division of Paediatrics, University Medical Centre Maribor, 2000 Maribor, Slovenia

**Keywords:** stroke, vascular cognitive impairment, post-stroke depression, anxiety, fatigue, anhedonia, cluster analysis, neuropsychiatric symptoms

## Abstract

*Background and Objectives*: Stroke is widely recognised for its physical consequences. However, cognitive and emotional impairments, such as depression, anxiety, and vascular cognitive impairment (VCI), are often under-recognised and under-treated. Our study aimed to identify and characterise cognitive and emotional sequelae in patients hospitalised for acute ischemic stroke. *Materials and Methods*: We conducted a cross-sectional study involving 73 patients within seven days of an acute ischemic stroke. Patients were assessed using the National Institutes of Health Stroke Scale (NIHSS), modified Rankin Scale (mRS), Montreal Cognitive Assessment (MoCA), Hachinski Ischemic Score (HIS), and the Clinical Assessment of Depression (CAD) questionnaire, which includes four subscales (Depressed Mood (DM), Anxiety/Worry, Disinterest, and Physical Fatigue). K-means clustering was applied to ten standardised clinical and psychometric variables. In addition, multiple linear regression was performed to determine independent predictors of cognitive and affective outcomes, with MoCA and CAD-DM as dependent variables. *Results*: Three distinct patient profiles emerged: (1) Mild Impairment Profile, characterised by minimal cognitive or emotional symptoms; (2) Depressive Profile, marked by elevated emotional symptom scores despite mild physical impairment; and (3) Vascular Cognitive Impairment Profile, comprising older patients with the most severe cognitive and functional deficits. ANOVA confirmed significant differences between groups in NIHSS, mRS, MoCA, HIS, and CAD scores, but not for age or education. Linear regression revealed that older age (β = –0.10, *p* = 0.012) and higher NIHSS at discharge (β = –0.72, *p* = 0.020) predicted lower MoCA scores, whereas years of education (β = 0.58, *p* = 0.013) predicted better cognition (R^2^ = 0.29). No demographic or clinical factors predicted depressive symptoms (all *p* > 0.29). *Conclusions*: Our study highlights the heterogeneity of post-stroke outcomes. Neuropsychiatric impairments may be present even in patients with minimal physical deficits and require targeted evaluation and management.

## 1. Introduction

Stroke is the leading cause of permanent disability worldwide [1]. While stroke is often considered in terms of its physical symptoms, many stroke survivors suffer from long-term cognitive and psychological consequences [2,3]. Changes in mood and behaviour, with depression, anxiety, and vascular cognitive impairment (VCI) emerging as some of the most common neuropsychiatric outcomes, are often overlooked.

Cognitive and emotional sequelae are frequent complications following stroke, affecting up to 80% of patients and significantly impairing functional independence, quality of life (QoL), and long-term prognosis [4].

The reported prevalence of post-stroke depression ranges from approximately 18% to 31% depending on the study [5,6]. A recent meta-analysis found that post-stroke depression (PSD) affects approximately 27% of stroke survivors in general [7]. Prevalence is slightly higher when assessed by rating scales (29%) than by clinical interviews (24%). PSD is most common within the first month (30%), declining to 22% at 6–12 months. However, cumulative incidence within the first year post-stroke may reach 71% of cases, beginning within the first three months. Among those with early-onset depression, less than half of the patients recover.

Similarly, anxiety and VCI also exhibit significant rates, up to 50% of patients [5,6], underscoring the significance of these conditions in the recovery trajectory of stroke patients [8]. Risk factors include older age, pre-stroke depression, anxiety or cognitive impairment, stroke severity, and lesion location [9].

Although neuropsychiatric symptoms often coexist, they are influenced by various underlying mechanisms and are not solely determined by lesion locations, which do not always correlate with motor and sensory impairments [10,11]. Patients with lower scores on the modified Rankin Scale (mRS) and National Institutes of Health Stroke Scale (NIHSS) can exhibit severe neuropsychiatric sequelae, emphasising the complexity of lesions within brain networks [12].

Despite their prevalence, mood disorders and VCI after stroke remain under-recognised and under-treated in clinical practice, partly due to overlapping symptomatology with neurological sequelae. For example, symptoms like dysphasia, emotional lability, fatigue, and apathy may obscure or mimic core features of depression and anxiety, such as low mood, restlessness, or difficulty concentrating, complicating diagnosis and reducing the effectiveness of standard classification systems.

From a clinical perspective, it is essential to identify patients at increased risk in order to personalise their care and tailor our approach to meet their specific needs. Emotional disturbances can interfere with recovery, reduce motivation for rehabilitation, and negatively affect overall quality of life [3]. A recent umbrella review found that both pharmacological and non-pharmacological treatments for post-stroke depression can effectively reduce depressive symptoms, improve patient adherence to rehabilitation, and enhance neurological recovery [13]. In addition, post-stroke cognitive impairment, depression, and other mood disorders are associated with lower quality of life [14,15]. Interestingly, low quality of life in those patients may persist for months and regardless of the physical recovery [14,15].

This highlights the critical importance of integrated assessments of both cognitive and emotional health [5,6]. The latest guidelines from the European Stroke Organisation and the European Academy of Neurology suggest an active screening as part of a comprehensive assessment of stroke survivors [16]. Although the Montreal Cognitive Assessment (MoCA) is not a substitute for a comprehensive neuropsychological evaluation, we had previously validated the Slovenian version of the MoCA and the Hachinski Ischemic Scale (HIS) for screening for VCI in patients after transitory ischemic attack and stroke [17].

While neuropsychiatric outcomes are well documented, a critical gap remains in understanding whether certain subgroups of patients are more susceptible to such impairments based on demographic, vascular, or clinical profiles.

In the present study, we aimed to further explore the relationships between physical, cognitive, and emotional post-stroke sequelae. The critical gap addressed in our study is whether data-driven methods other than regression analysis can identify clinically meaningful patient subgroups in the acute phase, and which demographic or neurological factors predict outcomes. To address this complexity, we implemented a dual-method design. First, we used unsupervised cluster analysis to identify naturally occurring clinical subgroups based on cognitive and emotional symptomatology. This approach enabled us, similar to previous studies [18,19], to move beyond traditional screening thresholds and instead describe data-driven phenotypes; e.g., predominantly a cognitively impaired group, an emotionally impaired group, and a relatively preserved group—thus capturing the heterogeneity of post-stroke neuropsychiatric profiles.

Second, we applied linear regression modelling to examine whether demographic (age, education), vascular, and clinical factors independently predicted the severity of cognitive and emotional symptoms. Linear regression allows for the quantification of each factor’s contribution, offering practical insights into modifiable risk indicators.

By integrating these two complementary methods, our study provides both a syndromic and a mechanistic understanding of cognitive and emotional disturbances in the acute post-stroke phase. These findings may inform the design of personalised medicine, improve patient care, and help plan health care resources.

## 2. Materials and Methods

### 2.1. Study Design, Setting, and Participants

This cross-sectional study included adult consecutive patients admitted with acute ischemic stroke to the Division of Neurology, University Medical Centre, between September 2024 and July 2025. All participants were native speakers and signed the informed consent form prior to the study.

The inclusion criteria were age >18 years, diagnosis of acute ischemic stroke, and ability to complete cognitive and affective assessments.

The exclusion criteria were severe physical disability following stroke (mRS > 4), visual impairment, or aphasia. Patients who refused to sign the informed consent form after being informed about the study were also excluded. This report follows the Strengthening the Reporting of Observational Studies in Epidemiology (STROBE) guidelines [20].

### 2.2. Variables, Data Sources, and Measurements

Sociodemographic data, patients’ age, gender, education, and prior medical history, were collected. Data were extracted from clinical records and entered into a study database. All patients were examined by an experienced neurologist, and their motor performance was evaluated with NIHSS [21] at admission and discharge and with mRS at discharge [22]. NIHSS includes 15 items that evaluate the level of consciousness, language, motor strength, sensory loss, visual fields, and coordination. Scores range from 0 to 42, with higher scores indicating more severe impairment. A similar but simpler scale is the mRS. Besides the degree of disability, it also measures the degree of dependence in the daily activities of post-stroke patients. Scores range from 0 (no symptoms) to 6 (death), providing an effective summary of functional outcome.

Cognitive and emotional status were assessed within seven days after the stroke. Cognition was assessed using the Slovenian version of MoCA and HIS [17]. The MoCA is a brief screening tool designed to detect VCI. It evaluates multiple cognitive domains: memory, attention, language, visuospatial skills, and executive function. The test has a maximum score of 30, with lower scores indicating cognitive impairment. The cut-offs vary among different countries and cultures, as well as underlying causes for cognitive impairment [23,24,25]. Although it is necessary to use validated cut-offs [17,26], MoCA has a high sensitivity and specificity, and it is widely used in both clinical and research settings, especially in stroke and dementia populations. For the Slovenian population, cut-off values are 25/26 points.

HIS is a clinical scale developed to differentiate between vascular and degenerative types of dementia [27]. It consists of a set of weighted questions related to the clinical history of vascular risk factors and neurological findings (e.g., sudden onset, stepwise deterioration, hypertension). Scores range from 0 to 18, with higher scores (typically ≥7) suggesting a vascular aetiology [26]. HIS is especially useful in identifying patients at risk for vascular cognitive impairment in stroke populations.

Emotional changes were evaluated using the self-reported Clinical Assessment of Depression (CAD) questionnaire, which consists of four subscales: Depressed Mood (DM), Anxiety/Worry (A/W), Diminished Interest (DI), and Cognitive and Physical Fatigue (CPF) [28]. Each subscale provides information on a specific patient’s emotional state, which allows us to understand their psychological condition. Raw subscale scores are standardised, and T scores more than 60 indicate mild, while scores above 80 indicate severe depression, anxiety, diminished interest/anhedonia, and fatigue.

### 2.3. Bias

To minimise selection bias, we approached all consecutive patients who met the eligibility criteria during the study period. Stroke was confirmed with brain CT or MRI. All patients received early rehabilitation in the ward. We reduced measurement bias by using validated assessment tools and standardised administration protocols. All evaluators received training and were experienced in utilising the clinical scales to ensure consistency and reliability.

### 2.4. Statistical Analysis

Basic statistical analyses were performed using jamovi (version 2.5), while advanced analyses were conducted in Python (version 3.12) with the statsmodels and scipy libraries.

#### 2.4.1. Sample Size

A formal a priori sample size calculation was not performed; instead, a post hoc power analysis was carried out based on the observed data. Overall, the study was sufficiently powered to detect moderate-to-large, between-group differences in cluster analysis.

To determine if our sample size was adequate for the linear regression model using MoCA and CAD as the dependent variable, we conducted a post hoc analysis to estimate the statistical power based on the observed effect sizes of significant predictors. We calculated the partial R^2^ values from the regression t-statistics, which yielded values of approximately 8% to 10% for age, education, and NIHSS at the discharge. These values were then converted into Cohen’s f^2^ indices, allowing us to compute the statistical power of 0.70–0.80 with *n* = 73.

To evaluate the adequacy of the available sample, achieved power was estimated from observed partial R^2^ values. Predictors explaining approximately 8–10% of unique variance in MoCA scores achieved a power of 0.70–0.80 with *n* = 73, indicating sufficient sensitivity for medium effects but limited power for smaller effects in the sample size.

Detailed calculations and results are presented in Appendix A.1.

#### 2.4.2. Cluster Analysis

**Figure A1:**
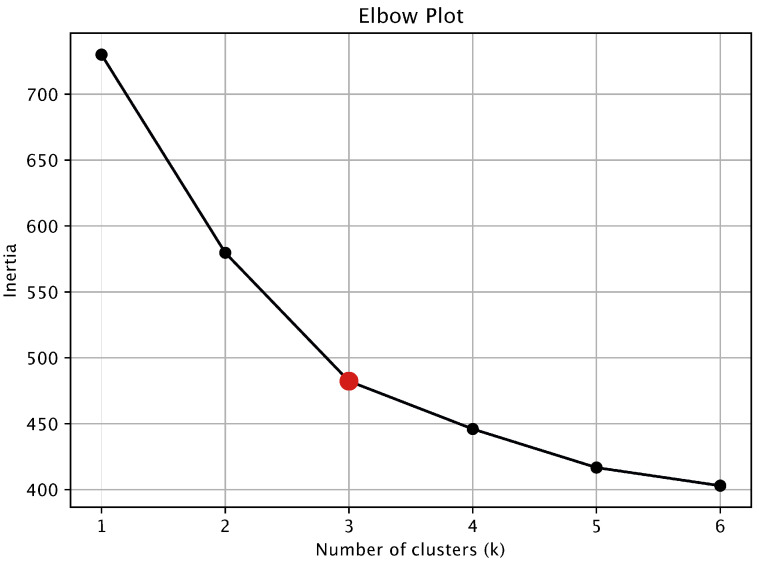
Elbow plot for K-means cluster analysis of cognitive, affective, and clinical variables. Red circle indicates optimal number of clusters.

K-means clustering was applied to the standardised dataset (z-scores). Ten clinically relevant variables (age, education, NIHSS at admission and discharge, mRS at discharge, MoCA, HIS, CAD-DM, CAD-A/W, CAD-DI, and CAD-CPF) were included in the clustering analysis. The optimal number of clusters (k = 3) was determined using the elbow method based on within-cluster sum of squares (inertia); see Appendix A.2, Figure A1.

Following clustering, we calculated the cluster centroids and visualised the profiles of the three patient clusters across the ten input variables to identify distinct cognitive, emotional, ischemic, disability, and demographic patterns. To assess the relative contribution of each variable in defining cluster separation, feature importance was calculated using a random forest classifier trained to predict cluster membership. Importance scores were extracted and visualised to highlight the most discriminative predictors.

After identifying three patient groups using K-means clustering, we first conducted a global comparison of all continuous variables across clusters using Kruskal–Wallis test, as appropriate. For variables showing significant overall differences, post hoc pairwise comparisons between clusters were performed using the Mann–Whitney U test with Bonferroni correction. Statistical significance was set at *p* < 0.05. Descriptive statistics were calculated for all variables within each cluster.

#### 2.4.3. Linear Regression

To explore predictors of continuous cognitive and affective outcomes, we performed multiple linear regression analyses with the Montreal Cognitive Assessment (MoCA) and the total Clinical Assessment of Depression score (CAD_total) as dependent variables. Predictor variables were selected a priori based on clinical plausibility and included age, years of education, NIHSS at admission, NIHSS at discharge, mRS at discharge, and Hachinski Ischemic Score (HIS). All predictors were entered simultaneously using the enter method (forced entry), thereby estimating the independent contribution of each variable while controlling for the others.

Prior to analysis, continuous predictors were examined for normality, linearity, and multicollinearity. Variance inflation factors (VIFs) were <2 for all predictors, indicating no multicollinearity. Residual plots were visually inspected to confirm model assumptions of linearity and homoscedasticity. Statistical significance was set at *p* < 0.05 (two-tailed). Standardised regression coefficients (β), 95% confidence intervals, and *p*-values were reported for individual predictors, while model fit was expressed as R^2^.

### 2.5. Ethics

We conducted the present study in accordance with the Declaration of Helsinki, and the IRB of the University Medical Centre Maribor reviewed and approved the protocol UKC-MB-KME-67/22, 13 December 2022 and amendments UKC-MB-KME-45/25, 9 June 2025.

## 3. Results

### 3.1. Sampling and Demographic Data

Out of 400 patients admitted during the study period, 73 (41 men and 32 women) met the eligibility criteria, representing 18.3% of the screened population (Table 1). The mean age was 65.5 years (95% CI 62.09 to 69.00 years). Most patients finished high school, with a mean duration of formal education of 11.79 years. A total of 31 patients had lesions in the right hemisphere, 28 patients had lesions in the left hemisphere of the cerebrum, and 14 had lesions in the cerebellum.

Among vascular and lifestyle risk factors, atrial fibrillation was present in 8 patients (11.0%), while arterial hypertension was the most common comorbidity, affecting 50 patients (68.5%). A positive family history of cardiovascular disorders was reported by 46 patients (63.0%), and 19 patients (26.0%) reported a family history of dementia. In addition, 20 patients (27.4%) were active smokers at the time of stroke.

Most patients remained in the ward for two weeks. During this time, they began early rehabilitation and received physiotherapy.

When considering cut-offs for MoCA 24/25 and CAD domain thresholds (T ≥ 60, mild clinical risk), the incidence of patients with some form of VCI was 68.5%, depression 17.8%, anxiety 26.0%, fatigue 15.1%, and loss of interest (anhedonia) 15.1%.

**Figure A2:**
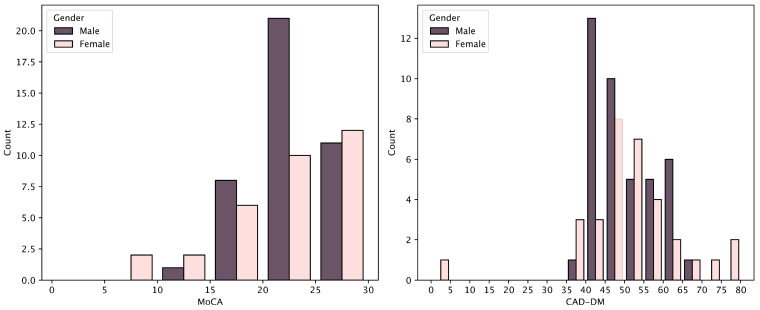
Distribution of Montreal Cognitive Assessment (MoCA) and Clinical Assessment of Depression (CAD) standardised depression (DM) scores by sex.

Distribution of MoCA and CAD standardised DM scores by sex is presented in Appendix A, Figure A2.

### 3.2. Patients’ Symptom Profiles

After performing K-means clustering analysis, three distinct clusters emerged: Cluster 1—Mild Impairment profile (MIP) comprised patients with mild physical impairment and minimal cognitive or emotional symptoms; Cluster 2—Depressive Profile (DP) included patients with a pronounced depressive profile; and Cluster 3—Vascular Cognitive Impairment (VCI) profile was characterised by patients with predominant cognitive deficits consistent with vascular cognitive impairment.

**Figure 1:**
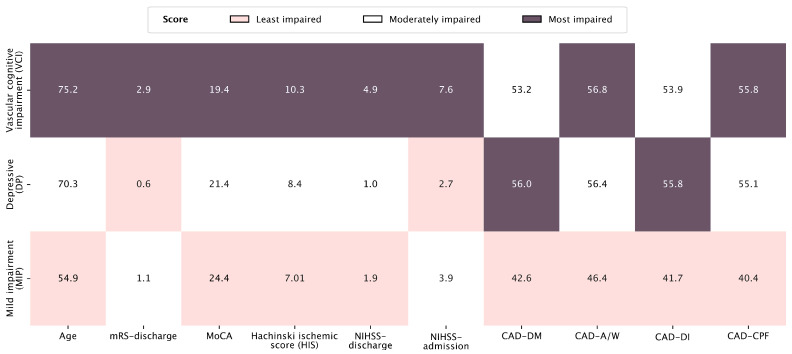
Cluster profiles based on cognitive, emotional, ischemic, disability, and demographic variables. Scores represent cluster centroids. NIHSS—National Institutes of Health Stroke Scale; mRS—modified Rankin Scale; MoCA—Montreal Cognitive Assessment; HIS—Hachinski Ischemic Score; CAD-DM—Cognitive and Affective Disorders scale—Depressed Mood subscale; CAD-A/W—Anxiety/Worry subscale; CAD-DI—Disinterest subscale; CAD-CPF—Cognitive and Physical Fatigue subscale.

The MIP group included 28 patients (38% of the sample). Patients had minimal emotional symptoms, mild neurological deficits, and relatively preserved cognitive functions (Figure 1). These patients were the youngest, with a mean age of 54.9 years. They expressed only mild cognitive symptoms and had the highest MoCA scores, of 24.4 points on average. They expressed only minimal post-stroke depression, anxiety, fatigue, and anhedonia, obtaining the lowest mean scores on all CAD subscales. In addition, they had the best physical and functional outcomes, with minimal scores on the NIHSS (mean: 3.9 at admission, 1.9 at discharge) and mRS (mean: 1.1 at discharge).

The second cluster (DP profile) was a similar size and included 28 patients (38%). Patients were older than those in cluster 1 but younger than those in cluster 3, with a mean age of 70.3 years. These patients demonstrated mild physical and functional neurological deficits at admission and discharge, with a mean NIHSS of 2.7 at admission (1.0 at discharge), and the lowest mean mRS of 0.6 among all clusters. However, they had prominent emotional disturbances and had the highest mean scores on the CAD subscales Depressed Mood (56.0) and Diminished Interest (55.8) and very high scores on Anxiety/Worry (56.4) and Fatigue (55.1).

The patients with prominent VCI consisted of the third cluster. In this group, 17 patients (23%) were included. The VCI group was characterised by the highest mean age (75.2 years), the most severe neurological impairment at admission and discharge (mean NIHSS 7.6 at admission, 4.9 at discharge), and the most significant functional decline (mRS 2.9 at discharge). Their cognitive performance was the worst among all three groups. The mean MoCA score was 19.4, consistent with moderate cognitive impairment. Although their emotional symptoms were similarly pronounced as in the DP group, they expressed the most pronounced physical and cognitive fatigue in the CAD-CPF subscale among all three groups (mean score 55.8) and anxiety (mean score 56.8). In addition, all other CAD subscale mean scores were significantly lower than those of the patients in the cluster 1—MIP profile (Figure 1).

Cluster profiles with mean values are summarised in Figure 1.

**Figure 2:**
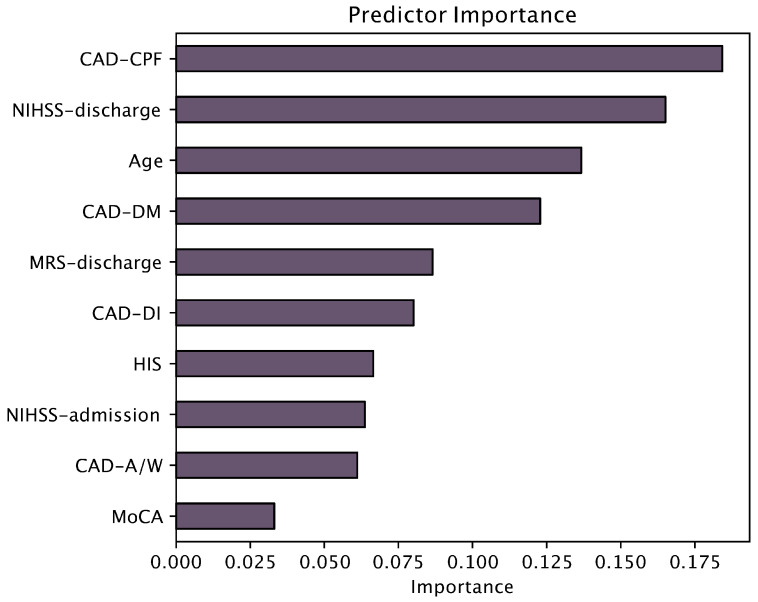
Cluster assignment based on random forest analysis. NIHSS—National Institutes of Health Stroke Scale; mRS—modified Rankin Scale; MoCA—Montreal Cognitive Assessment; HIS—Hachinski Ischemic Score; CAD-DM—Cognitive and Affective Disorders scale—Depressed Mood subscale; CAD-A/W—Anxiety/Worry subscale; CAD-DI—Disinterest subscale; CAD-CPF—Cognitive and Physical Fatigue subscale.

The most influential predictors, as shown in the random forest plot (Figure 2), were the CAD-CPF scores, the NIHSS score at discharge, age, and the CAD-DM. On the other hand, HIS contributed four times less to clustering, while MoCA six times less, than the most influential predictor.

After conducting ANOVA, we found very significant differences (*p* < 0.001) for all variables between at least two clusters (Table 1). The only exception was years of education, for which we did not find statistically significant differences.

**Figure 3:**
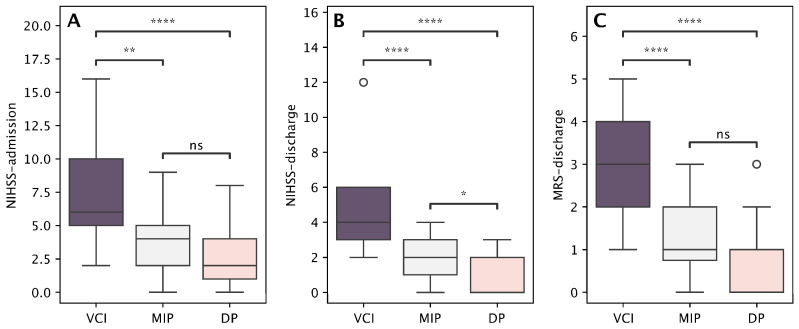
Between-cluster comparisons of neurological impairment and functional outcome measures, namely NIHSS at admission (**A**), NIHSS at discharge (**B**), and mRS at discharge (**C**), among the three identified profiles: Vascular Cognitive Impairment (VCI) profile, Depressive Profile (DP), and Mild Impairment Profile (MIP). Asterisks denote significance levels: * *p* < 0.05, ** *p* < 0.01, **** *p* < 0.0001; “ns” indicates non-significant differences (Bonferroni-adjusted).

In the end, we performed post hoc analysis with a Bonferroni-adjusted pairwise Mann–Whitney U test. The most physically impaired patients were those with a VCI profile. They exhibited significantly higher NIHSS scores compared to the other two groups, both at admission and at discharge (Figure 3A,B). This finding was similarly observed when comparing patients in the DP group to those in the MIP group. Furthermore, compared to the other two groups, patients with the VCI profile expressed a significant functional impairment, reflected in the highest mRS scores at discharge. There were no significant differences in functional disability at discharge between DP and MIP (Figure 3C).

**Figure 4:**
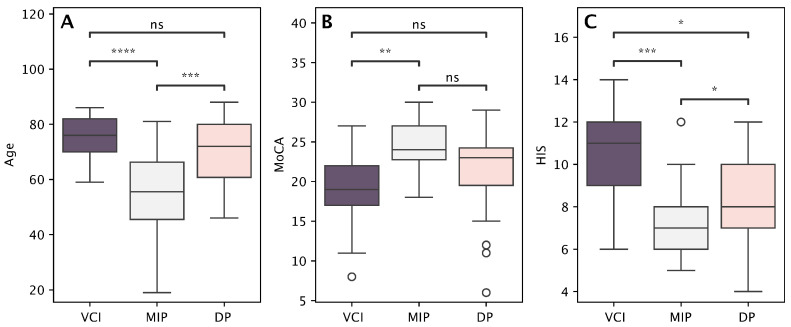
Between-cluster comparisons of demographic and cognitive–vascular characteristics, namely age (**A**), HIS (**B**), and MoCA (**C**), among the three identified profiles: Vascular Cognitive Impairment (VCI), Depressive Profile (DP), and Mild Impairment Profile (MIP). Asterisks denote significance levels: * *p* < 0.05, ** *p* < 0.01, *** *p* < 0.001, **** *p* < 0.0001; “ns” indicates non-significant differences (Bonferroni-adjusted).

Patients in the MIP group were significantly younger than patients with other profiles. However, we did not find any significant differences between VCI and DP groups (Figure 4A). The vascular burden, measured with HIS, was significantly higher in patients with VCI and DP than in the MIP group. However, there were no statistically significant differences between the VCI and DP groups (Figure 4B). Additionally, the MoCA score was significantly lower in the VCI group than in the MI group, though no significant differences were observed between the VCI and DP groups (Figure 4C).

**Figure 5:**
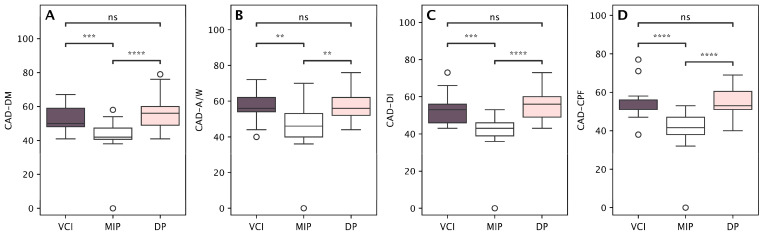
Between-cluster comparisons of emotional and cognitive symptom domains assessed with the Clinical Assessment of Depression (CAD) subscales questionnaire among the three identified profiles: Vascular Cognitive Impairment (VCI), Depressive Profile (DP), and Mild Impairment Profile (MIP). CAD Depressed Mood subscale (**A**); Anxiety/Worry subscale (**B**); Diminished Interest subscale (**C**); Cognitive and Physical Fatigue subscale (**D**). Asterisks denote significance levels: ** *p* < 0.01, *** *p* < 0.001, **** *p* < 0.0001; “ns” indicates non-significant differences (Bonferroni-adjusted).

Patients in the VCI profile group and the DP group reported significantly more depressive symptoms than patients in the MIP group. Similar results were found for anxiety, loss of interest, and cognitive and psychological fatigue. All CAD subscale scores were significantly elevated for both the VCI and DP groups in comparison to the MI group, with no statistically significant differences between the VCI and DP groups (Figure 5A–D).

### 3.3. Linear Regression

We used MoCA as a dependent factor in multiple linear regression and identified several significant predictors of cognitive outcome (Table 2). Older age was associated with lower MoCA scores (β = −0.10, *p* = 0.012), while more years of education predicted better performance (β = 0.58, *p* = 0.013). Higher NIHSS scores at discharge were independently associated with lower MoCA performance (β = −0.72, *p* = 0.020). Together, these predictors explained approximately 29% of the variance in MoCA scores, indicating moderate explanatory power. Other variables, including NIHSS at admission, mRS at discharge, and HIS, were not statistically significant.

In contrast, the regression model with CAD-DM as the dependent variable did not yield any significant predictors (all *p* > 0.29), suggesting that emotional symptoms could not be explained by demographic or stroke-related clinical measures in this cohort (Table 3).

## 4. Discussion

Our findings contribute to the current understanding of cognitive and emotional outcomes following stroke by highlighting the heterogeneity of symptom presentation and the significant influence of methodological approaches on prevalence estimates.

### 4.1. Patients’ Profiles Following Stroke

While previous studies typically report cognitive and emotional sequelae as collective post-stroke outcomes [29,30], our data-driven approach identified three distinct patient profiles characterised by predominantly cognitive, emotional, or mild combined impairment. This subgroup delineation underscores the clinical diversity within stroke cohorts and suggests the need for tailored assessment and interventions.

In our cluster model, fatigue emerged as one of the most influential predictors, particularly in distinguishing patients with a high emotional and cognitive burden. This is in accordance with previous studies. The American Association describes post-stroke fatigue as a multidimensional condition encompassing physical and cognitive components [31]. The reported incidence of post-stroke fatigue varies widely, ranging from 22% to 70% depending on the study population and the assessment tools used [32,33]. Fatigue has been consistently shown to correlate strongly with other neuropsychiatric symptoms, including depression, anxiety, cognitive impairment, and physical disability. In particular, recent evidence indicates that post-stroke patients with coexisting depression, anxiety, poor sleep quality, and reduced self-perceived recovery report significantly greater levels of both physical and cognitive fatigue, as measured by the Modified Fatigue Impact Scale (MFIS) [32]. These findings suggest that fatigue is not only a common symptom but also an important factor contributing to the broader post-stroke neuropsychiatric profile. Patients in both VCI and DP expressed significantly more fatigue than patients with mild impairment. From a clinical perspective, it may be important to recognise and address fatigue early, as it may improve rehabilitation compliance and enhance long-term functional outcomes.

An interesting result from our analysis is that significant depressive and cognitive impairments may also affect patients with relatively favourable physical outcomes. Specifically, many patients in the depression-predominant group exhibited only minor physical impairment, and most patients in the VCI group maintained ambulation capability at discharge (Table 1). Our findings support recent literature showing that depressive symptoms frequently manifest even among stroke survivors who received up-to-date treatment and have minimal physical deficits [34]. This is in contrast to older data where post-stroke depression was mainly found in patients with greater functional impairment and emotional distress [35]. Consequently, our findings stress the importance of assessing cognitive and emotional sequelae independently of physical function during stroke rehabilitation and follow-up.

Our study identifies clear risk factors for VCI, including greater vascular burden, older age, and more pronounced physical impairment. Given that patients within the VCI cluster were significantly older, it is plausible that advanced age partly influenced performance on cognitive screening tests such as the MoCA [17]. Nevertheless, our results confirm the critical role of vascular pathology in cognitive decline. Recent research, as well as our previous work, indicates that vascular risk factors, including white matter lesions, hypertension, and diabetes, account for substantial proportions of dementia incidence globally, underscoring the central importance of vascular mechanisms in post-stroke cognitive outcomes [17,36].

Patients in our cohort primarily categorised with VCI exhibited significantly lower scores on cognitive measures compared to those predominantly affected by depression. Previous studies suggest that emotional disturbances can negatively affect cognitive test performance. Patients experiencing major depressive disorder were likely to demonstrate significant cognitive impairment [29]. Therefore, it is possible that the lower MoCA scores in the second group were not only due to VCI but also due to depressive symptoms (Table 2).

Moreover, anxiety, fatigue, and anhedonia did not significantly differ between cognitively and emotionally impaired groups. This implies the existence of two distinct predominant sequelae post-stroke—VCI and depression—rather than a generalised neuropsychiatric dysfunction.

When interpreting our findings, it is important to note that clustering was based on the combined pattern of multiple variables, while post hoc comparisons evaluated each variable individually. Not all variables showed significant between-group differences, which reflects the distinction between unsupervised clustering and inferential statistics. Cluster analysis captures multivariate patterns even in smaller samples, whereas the Kruskal–Wallis and Mann–Whitney U tests assess differences in single variables. Significant differences in NIHSS, mRS, HIS, MoCA, and CAD subscores—especially between the VCI/DP and MIP groups—support the validity of the identified profiles, even if some variables were not independently significant.

From the clinical perspective, it is worth noting that cognitive and emotional sequelae are dynamic. Chen et al. [18] found with their cluster analysis that anxiety was the predominant emotional symptom in the acute post-stroke phase. However, in the following six months, anxiety was replaced with depression in some patients. Interestingly, a recent long-term study on the temporal progression of fatigue demonstrated that fatigue remained relatively stable across time [37]. However, it was still associated with other emotional, cognitive, and functional post-stroke outcomes.

We evaluated patients within seven days post-stroke. Most of the patients had early rehabilitation and subthreshold emotional sequelae and relatively mild physical and functional impairments at discharge. Therefore, it is possible that the number of patients in clusters as well as the cluster profiles would change in a longitudinal observation, especially for patients with poorer physical and emotional outcomes.

### 4.2. Incidence of Neuropsychiatric Symptoms

Another important observation from our study relates to the influence of methodological approaches on the estimated prevalence of cognitive and emotional sequelae post-stroke. Utilising conventional screening thresholds, such as a MoCA score below 25 [17] or CAD-E domain-specific criteria [28], we observed notable prevalence rates among the patient population. Specifically, 68.5% of patients were identified as having some form of VCI, highlighting significant cognitive challenges possibly linked to vascular conditions. Additionally, 17.8% of patients exhibited symptoms of depression, suggesting a considerable emotional burden that could influence overall cognitive function. The data also revealed that 26.0% of patients experienced anxiety, which can further complicate cognitive assessments and treatment approaches. Furthermore, 15.1% reported physical or psychological fatigue, a common complaint that might affect daily functioning, while another 15.1% showed signs of anhedonia or a loss of interest in previously enjoyable activities.

In contrast, when we compared these results to those obtained through data-driven methodologies like unsupervised clustering, we found narrower but more clinically meaningful syndromic profiles.

It may seem paradoxical that the incidence of cognitive impairment decreased from 68.5% using conventional screening criteria to 23% using clustering methods, and the prevalence of depression increased from 17.8% to 38% (Table 1). However, these findings highlight that cognitive or emotional impairment should not rely solely on test results but should be interpreted within a broader context.

We emphasise that this difference does not represent an actual decrease in the incidence of cognitive impairment or an increase in depression but rather reflects the methodological distinction between single-variable screening and multidimensional classification. The test scores provide a unidimensional approximation based on a validated cut-off score. In contrast, the clustering approach integrates cognitive, affective, and clinical features simultaneously, allowing the identification of symptoms that extend beyond VCI or depression alone.

Within this framework, the VCI or DP should not be interpreted strictly as “major cognitive impairment” or “major depression,” but rather as a broader emotional phenotype characterised by the co-occurrence of cognitive or depressive symptoms with related features such as anxiety, loss of interest, and fatigue. For example, patients who do not reach the CAD depression threshold may nevertheless cluster with individuals who have high depressive score, because they share overlapping cognitive, emotional, and clinical characteristics. This explains why the cluster prevalence is higher than the isolated depression rate and why clustering is valuable for revealing patterns of comorbidity and subthreshold symptom burden that may otherwise be overlooked.

Variability in reported incidence rates across studies can also be attributed to several factors, including demographic and clinical heterogeneity, as well as differences in the cut-off scores used for assessing cognitive and affective impairments on specific standardised tests. For instance, the cut-off scores for MoCA and VCI vary significantly across countries, with some setting the threshold as low as 20 points and others as high as 26 points [38]. These variances may reflect social differences in culture, language, or the educational background of populations.

Furthermore, the discrepancies in reported incidence rates for depression and other emotional sequelae also depend on numerous factors, such as the age of the individuals being studied, the time interval since the initial assessment, and the specific methods used for evaluation, which can include self-reported questionnaires, structured interviews, or clinical assessments. For example, younger populations may report different emotional experiences compared to older adults, and those evaluated shortly after a traumatic event might show higher incidence rates than those assessed months or years later [7,11].

Although only 23% of patients were classified into the VCI cluster through data-driven analysis, it is important to note that a substantial proportion of patients still exhibited some degree of vascular-related cognitive or emotional impairment. Many of these patients had elevated HIS or borderline MoCA results, suggesting underlying vascular contributions that may not have manifested as overt cognitive syndromes but could still interfere with attention, executive function, and motivation—key factors in rehabilitation success. Thus, even in the absence of full-blown VCI, subclinical vascular impairment may compromise engagement and performance during recovery.

Clinically, this broader definition is important because subthreshold cognitive and emotional symptoms are known to affect recovery, adherence to rehabilitation, and quality of life after stroke. By grouping these patients into a coherent profile, clustering provides a more holistic representation of emotional sequelae better aligned with real-world clinical presentation. Thus, the clustering analysis does not increase or decrease the incidence but rather contextualises it within a multidimensional framework, even when single test scores do not reach categorical diagnostic thresholds.

Recognising these subtler forms of dysfunction is crucial for designing supportive, tailored rehabilitation plans at the individual level and effectively addressing cognitive and emotional barriers in post-stroke patients at an early stage.

### 4.3. Clinical Implementations of Predictive Factors for Vascular Cognitive Impairment and Depression

While clustering provides the necessary broadness to understand the stroke sequelae at the cohort level, it is important to address patients’ individual needs in clinical settings. Therefore, we performed linear regression to explore the predictors of cognitive performance and depressive symptoms in the acute phase after ischemic stroke.

The model for MoCA explained a moderate proportion of variance and highlighted three significant predictors. Age emerged as a robust negative determinant (Table 3), indicating that each additional year was associated with a slight but meaningful decline in MoCA score. As expected, years of education positively influenced cognitive outcomes. Similar results were observed in our previous study examining patients with VCI [17] and in other studies using MoCA [39]. These results support the idea of the protective role of cognitive reserve in acute stroke.

An important observation is the reversible relationship between physical and cognitive impairment. Due to collinearity between NIHSS and mRS, we limited our model to the NIHSS only. Although NIHSS at admission was not a significant predictor of MoCA performance in our cohort, a higher NIHSS at discharge was independently associated with lower MoCA performance (Table 3). Each additional point on the NIHSS at discharge was associated with a decrease of about 0.7 points on the MoCA. In a clinical setting, this may indicate that even small increases in residual neurological severity translate into measurable reductions in cognitive performance. Since the MoCA cut-off for impairment is 25, a difference of only 2–3 NIHSS points can shift a patient from normal to impaired cognition. Our model emphasises the connection between residual neurological severity and cognitive impairment, affirming findings from previous studies [40]. Furthermore, it demonstrates that subtle neurological deficits following a stroke in our cohort are significant and carry cognitive implications.

Other variables, including HIS, depressive symptoms, and hemispheric lateralisation, were not significant contributors in the present model (Table 3). Although some reports indicate that the side of the lesion may influence MoCA due to the test’s domain-specific sensitivity [41], this was not the case in our cohort. Similar results were found for vascular risk factors and depressive symptoms.

Taken together, these results emphasise that older age, lower education, and greater neurological deficits at discharge jointly shape acute post-stroke cognition.

In contrast, the regression model predicting depressive symptoms did not identify any significant predictors. Although age, education, HIS, NIHSS, MoCA, and lesion laterality were included, none reached statistical significance (all *p* > 0.29) (Table 3). This suggests that depressive symptom burden in the acute stage may not be directly explained by the demographic or clinical stroke characteristics captured in our cohort. Those results are similar to some previous studies [35,42]. For example, Shi et al., in their meta-analysis, did not find consistent results for risk factors, including sociodemographic variables [35]. On the other hand, in a similar acute post-stroke period, Yang et al. reported homocysteine, NIHSS, and the Activity of Daily Living Scale scores as independent predictors of early post-stroke depression [43]. There were several important differences between their retrograde study from our perspective. First, patients in their sample were more physically impaired with a higher NIHSS score, and the prevalence of depression was more than twice that of ours. Second, we assessed depression with the CAD, while Yange et al. used the Hamilton depression scale 17 and the Diagnostic and Statistical Manual of Mental Disorders. In addition, they included several laboratory tests and the Activity of Daily Living Scale score. However, other vascular risk factors, such as smoking, hypertension, and diabetes, as well as several serum markers, blood lipids, prealbumin, and fibrinogen, did not differ between post-stroke patients with and without depression. This is in accordance with our results, where HIS score was not a significant variable in a linear model.

Clinically, these results highlight the differential causes of cognitive and emotional outcomes. Cognitive performance may be partly predicted by established risk factors such as age, education, and stroke severity, while acute depressive symptoms appear more idiosyncratic.

They may be likely influenced by unmeasured psychosocial, biological, or premorbid factors. To better understand this, we need larger case controls and longitudinal studies. In a clinical setting, it is necessary to personalise our approach to the patients and actively screen for mood disturbances after stroke, as they cannot be identified from clinical or demographic variables alone.

### 4.4. Limitations

Our study also has its limitations. We included patients with only minor physical impairments, which may mean that severely affected individuals could display more emotional or cognitive symptoms. However, patients with aphasia would not be able to complete the MoCA assessment. Another limitation is the cross-sectional design and use of self-reported scales for the evaluation of emotions in our study. Due to the cross-sectional design, causality cannot be inferred, and results should be interpreted as associations rather than causal relationships. When interpreting our results, we must consider that patients were evaluated within seven days post-stroke. Our sample was large enough to detect medium to large differences. Therefore, it is possible that other variables would be recognised as significant in larger samples, where smaller effects would be found statistically significant. A longitudinal study with a comparison of cognitive and emotional burden between different time intervals could provide additional insights into cognitive and emotional recovery. In addition to the limitations already noted, residual confounding from unmeasured variables such as comorbidities, medication use, or premorbid psychiatric status may have influenced our findings.

## 5. Conclusions

Our study highlights the often neglected cognitive and emotional post-stroke impairments that can arise even when mild physical disabilities persist. The sequelae associated with stroke are heterogeneous and extend beyond motor deficits. Using cluster analysis, we identified three clinically meaningful profiles: a younger group with mild impairment, a depressive profile characterised by marked emotional burden despite minimal functional and mild cognitive deficits, and a vascular cognitive impairment profile associated with older age, cognitive decline, fatigue, and residual neurological severity.

Despite that, patients in our cohort displayed one of three clinically distinctive profiles, and mild to moderate cognitive and emotional symptoms were still evident across all three groups. These impairments are clinically significant and may disrupt the neurorehabilitation process. Furthermore, they may be easily overlooked, especially when the physical and psychological deficits appear mild. Our linear regression model identified three risk factors for vascular cognitive impairment: age, education, and NIHSS at discharge. However, we could not explain depression symptoms with demographic or stroke-related variables. This may suggest that early emotional reactions may reflect psychosocial adjustment as much as neuropsychiatric pathology.

Clinically, these findings highlight the importance of systematic, multidimensional patient assessment. Therefore, post-stroke patients require regular and comprehensive physical and psychological evaluations and a comprehensive approach to treatment that includes the full spectrum of patient symptoms.

Particular attention should be paid to older, less educated, and more physically impaired patients with fatigue, depression, and anxiety. Emotional symptoms may be present even in patients with good functional recovery and can hinder rehabilitation.

Recommendations for clinical practice should be regarded as hypotheses for further evaluation. Further longitudinal, multicentric studies are necessary to understand the long-term patterns of these impairments and for the development of personalised therapeutic strategies for both physical and cognitive rehabilitation.

## Figures and Tables

**Table 1:** Clinical, cognitive, and emotional characteristics of the patients.

	Vascular Cognitive Impairment Profile (*n* = 17)		Depressive Profile (*n* = 28)		Mild Impairment Profile (*n* = 28)		*p*-Value *
Incidence	23%		38%		38%		
	Mean	SD	mean	SD	mean	SD	
Age	75.2	8.2	70.3	11.5	54.9	14.6	<0.001
Years of education	11.4	2.7	11.5	2.4	12.4	2.0	ns
NIHSS at admission	7.6	4.4	2.7	2.1	3.9	2.3	<0.001
NIHSS at discharge	4.9	2.3	1.0	1.2	1.9	1.2	<0.001
mRS at discharge	2.9	1.1	0.6	0.8	1.1	0.9	<0.001
MoCA	19.4	5.1	21.4	5.3	24.4	3.1	0.020
HIS	10.3	2.3	8.4	1.9	7.1	1.7	<0.001
CAD—DM	53.2	7.5	56.0	9.1	42.6	9.8	<0.001
CAD—A/W	56.8	7.6	56.4	8.0	46.4	12.4	<0.001
CAD—DI	53.9	9.9	55.8	7.9	41.7	9.6	<0.001
CAD—CPF	55.8	9.6	55.1	7.6	40.4	9.8	<0.001

NIHSS—National Institutes of Health Stroke Scale; n – sample size; mRS—modified Rankin Scale; MoCA—Montreal Cognitive Assessment; HIS—Hachinski Ischemic Score; CAD-DM—Cognitive and Affective Disorders scale—Depressed Mood subscale; CAD-A/W—Anxiety/Worry subscale; CAD-DI—Disinterest subscale; CAD-CPF—Cognitive and Physical Fatigue subscale. All CAD scores are T scores, necessary for clinical evaluation (T ≥ 60 indicate mild emotional impairment). * Kruskal-Wallis test between clusters.

**Table 2:** Linear regression model for predicting post-stroke cognitive impairment. The Montreal Cognitive Assessment (MoCA) was a dependent variable.

Predictor	Estimate	SE	t	*p*
Intercept ^a^	22.5990	4.6902	4.818	<0.001
Age (years)	−0.1000	0.0386	−2.592	0.012
CAD-DM T score	−0.0112	0.0462	−0.243	0.809
Education (years)	0.5847	0.2282	2.562	0.013
HIS	−0.1564	0.2451	−0.638	0.526
NIHSS at admission	0.1988	0.1950	1.019	0.312
NIHSS at the discharge	−0.7180	0.3008	−2.387	0.020
Lesion location				
Left hemisphere—cerebellum	1.6140	1.4718	1.097	0.277
Right hemisphere—cerebellum	2.5769	1.5239	1.691	0.096

^a^ Represents reference level; NIHSS—National Institutes of Health Stroke Scale; MoCA—Montreal Cognitive Assessment; HIS—Hachinski Ischemic Score; CAD-DM—Cognitive and Affective Disorders scale—Depressed Mood subscale.

**Table 3:** Linear regression model for predicting post-stroke depression. The Cognitive and Affective Disorders scale—Depressed Mood subscale (CAD—DM) T score was a dependent variable.

Predictor	Estimate	SE	t	*p*
Intercept ^a^	39.8294	13.930	2.8592	0.006
Age (years)	0.1143	0.109	1.0524	0.297
Education (years)	0.2460	0.647	0.3804	0.705
HIS	0.4714	0.662	0.7124	0.479
NIHSS at admission	0.0477	0.531	0.0898	0.929
NIHSS at the discharge	−0.4314	0.847	−0.5096	0.612
MoCA	−0.0819	0.338	−0.2427	0.809
Lesion location				
Left hemisphere—cerebellum	−2.5795	4.001	−0.6448	0.521
Right hemisphere—cerebellum	−0.9311	4.207	−0.2213	0.826

^a^ Represents reference level; NIHSS—National Institutes of Health Stroke Scale; MoCA—Montreal Cognitive Assessment; HIS—Hachinski Ischemic Score; MoCA—the Montreal Cognitive Assessment.

## Data Availability

Data are unavailable due to privacy and ethical restrictions.

## Abbreviations

The following abbreviations are used in this manuscript:

NIHSS	National Institutes of Health Stroke Scale
mRS	modified Rankin Scale
MoCA	Montreal Cognitive Assessment
HIS	Hachinski Ischemic Score
CAD-DM	Cognitive and Affective Disorders scale—Depressed Mood subscale
CAD-A/W	Cognitive and Affective Disorders scale—Anxiety/Worry subscale
CAD-DI	Cognitive and Affective Disorders scale—Disinterest subscale
CAD-CPF	Cognitive and Affective Disorders scale—Cognitive and Physical Fatigue subscale
VCI	Vascular Cognitive Impairment
DP	Depressive Profile
MIP	Mild Impairment Profile

## Appendix A

### Appendix A.1. Post Hoc Sample Size Power Analysis

With 73 participants divided into three clusters (*n* = 17, 28, 28), this study achieved excellent power (>0.99) to detect the large observed between-group differences in NIHSS-discharge (η^2^ = 0.51, f = 1.01, power = 1.00), mRS-discharge (η^2^ = 0.47, f = 0.95, power = 1.00), and CAD-DM (η^2^ = 0.32, f = 0.69, power = 1.00). The analysis also had high power (≈0.92) for medium-to-large effects, as seen in MoCA (η^2^ = 0.17, f = 0.44). In contrast, variables with smaller effect sizes, such as years of education (η^2^ = 0.04, f = 0.19), were underpowered (≈0.29). Overall, the study was sufficiently powered to detect moderate-to-large between-group differences, but small effects should be interpreted with caution.

For the MoCA and CAD linear regression, predictors with partial R^2^ of 0.08–0.10 (age, education, NIHSS at discharge) achieved power of approximately 0.65–0.80 with the current sample size of *n* = 73. This indicates that our study was sufficiently powered to detect large-to-moderate associations but had limited power for smaller effects.

### Appendix A.2. Cluster Analysis

The elbow method was used to analyse the within-cluster sum of squares in order to determine the optimal number of clusters. A noticeable inflexion point occurs at k = 3, which supports choosing a three-cluster solution that provides the best balance between model fit and simplicity.

### Appendix A.3. Distributions of MoCA and CAD Scores

Histograms depict the distribution of Montreal Cognitive Assessment (MoCA) scores (left panel) and CAD standardised scores (right panel) among male and female post-stroke patients. The MoCA scores demonstrated a clustering around the clinical cut-off of 25 for both sexes, indicating that a substantial proportion of individuals exhibit cognitive impairment.

Furthermore, CAD scores were observed to be significantly higher on average in males; however, there was notable overlap in the distributions between genders, suggesting a degree of cognitive variability irrespective of sex.
